# The Antioxidant and Anti-Inflammatory Effects of *Quercus brantii* Extract on TNBS-Induced Ulcerative Colitis in Rats

**DOI:** 10.1155/2021/3075973

**Published:** 2021-01-07

**Authors:** Mahvash Alizade Naini, Shayan Mehrvarzi, Asal Zargari-Samadnejadi, Nader Tanideh, Mohammad Ghorbani, Amirreza Dehghanian, Maryam Hasanzarrini, Farnaz Banaee, Omid Koohi-Hosseinabadi, Cambyz Irajie, Aida Iraji

**Affiliations:** ^1^Gastroenterhepatology Research Center, Shiraz University of Medical Sciences, Shiraz, Iran; ^2^Department of Internal Medicine, Shiraz University of Medical Sciences, Shiraz, Iran; ^3^Student Research Committee, Shiraz University of Medical Sciences, Shiraz, Iran; ^4^Stem Cells Technology Research Center, Shiraz University of Medical Sciences, Shiraz, Iran; ^5^Health Sciences Research Center, School of Health, Torbat Heydariyeh University of Medical Sciences, Torbat Heydariyeh, Iran; ^6^Trauma Research Center, Shiraz University of Medical Sciences, Shiraz, Iran; ^7^Molecular Pathology and Cytogenetics Division, Department of Pathology, Shiraz University of Medical Sciences, Shiraz, Iran; ^8^Department of Internal Medicine, School of Medicine, Hamedan University of Medical Sciences, Hamedan, Iran; ^9^Department of Internal Medicine, School of Medicine, Shiraz University of Medical Sciences, Shiraz, Iran; ^10^Central Research Laboratory, Shiraz University of Medical Sciences, Shiraz, Iran; ^11^Laparoscopy Research Center, Shiraz University of Medical Sciences, Shiraz, Iran; ^12^Department of Medical Biotechnology, School of Advanced Medical Sciences and Technologies, Shiraz University of Medical Sciences, Shiraz, Iran; ^13^Medicinal and Natural Products Chemistry Research Center, Shiraz University of Medical Sciences, Shiraz, Iran

## Abstract

**Objectives:**

Ulcerative colitis is a common subtype of persistent inflammatory bowel disease with high morbidity consequences. Despite unknown definite pathogenesis, multiple anti-inflammatory medications are used for its treatment. Traditionally, *Quercus brantii* (QB), mostly available in the Middle East, has been used for gastrointestinal disorders. Other beneficial effects associated with QB include reduction of oxidative stress, inflammations, homeostatic instability, and improvement in clinical conditions.

**Materials and Methods:**

This experimental study was designed to assess the possible therapeutic effects of QB on UC and compare its effects with those of sulfasalazine. Of the 70 Wistar rats clustered in seven groups, ten received only alcohols and sixty were confirmed to be suffering from trinitrobenzene sulfonic acid- (TNBS-) induced colitis. Four groups received different dosages of QB extract via oral and rectal routes, one received sulfasalazine, and the other remaining two groups received nothing. The effects of QB were evaluated by assessing macroscopic and histologic scoring, measuring inflammatory mediators, and determining oxidative stress markers.

**Results:**

Comparing to the untreated TNBS-induced control groups, QB-treated groups showed a dose- and route-dependent improvement comparable with sulfasalazine. Treating rats with QB reduced the microscopic and macroscopic damage, decreased TNF-*α*, IL-6, NO, MPO activity, and MDA content, increased superoxide dismutase (SOD) activity, and reduced body weight loss.

**Conclusions:**

Our data recommended the anti-inflammatory and antioxidant effects of QB extract in a dose-dependent manner.

## 1. Introduction

Inflammatory bowel disease (IBD), with two major subtypes, namely, ulcerative colitis (UC) and Crohn's disease (CD), is a chronic condition associated with the inflammation of the alimentary tract and some extraintestinal manifestations [1, 2]. IBD patients are prone to acute and chronic complications, such as bleeding, toxic megacolon, primary sclerosing cholangitis (PSC), and colorectal cancer (CRC). Moreover, IBD has a significant impact on the quality of life with direct or indirect health care costs [3–5]. As a main subgroup of IBD, UC affects the large bowel of the gastrointestinal (GI) tract with a broad spectrum of acute and chronic clinical presentations. Although its exact etiology remains unknown, it has been suggested that genetic susceptibility and the activation of the mucosal immune system in response to bacterial antigens (intestinal IgG^+^ and anticommensal IgG) can play a role in causing this disease [6].

Epithelial damage, mucosal inflammation, and ulcerations are characterized in UC which are driven by activated cells of the immune system and altered T-cell mediated immunity. Also, the overproduction of circulating monocytes, mucosal macrophages, and gut microbiota has been reported to have a pathogenic effect on UC [7–10]. The activation of the immune system, T-lymphocytes, and *T* helper cells especially Th2 cells produces large amounts of cytokines and mediators which induce tissue damage and inflammatory responses in patients with UC [11].

Various studies confirmed that cytokines play an important role in the immunopathogenesis of UC. When the balance between proinflammatory and anti-inflammatory cytokines is disrupted, the release of proinflammatory cytokines (TNF, IL-1, IL-6, IL-12, and IL-23, etc.) increases, the tissue will damage, and UC disease progresses. During immunological dysregulation, tumor necrosis factor-*α* (TNF-a), known as an inflammatory mediator, secreted and promoted apoptosis which in return results in the production of IL-1 and IL-6 [12]. There is strong evidence that IL-6 increases in the serum and the intestinal mucosa of patients triggering and sustaining the intestinal inflammation response [13, 14]. The positive correlation between NO production and increased proinflammatory cytokine levels has been reported [15]. It seems that the high levels of NO as a free radical release in the mucosa cause tissue damage, exacerbate intestinal inflammation, and induce chronic UC.

Sulfasalazine is used to treat UC for more than 60 years. However, it just decreases symptoms such as fever, stomach pain, diarrhea, and rectal bleeding and does not cure this condition [16]. Sulfasalazine is a prodrug of 5-aminosalicylic acid (5-ASA) linked to sulfapyridine via an azo bond. Sulfasalazine is cleaved by azoreductases of intestinal bacterial, thus releasing sulfapyridine and 5-ASA. Active moiety in sulfasalazine is 5-ASA while the sulfapyridine acts as a carrier to prevent absorption of 5-ASA in the small bowel. 5-ASA directly contacts with colonic mucosa to suppress various proinflammatory pathways including both cyclooxygenase- and lipoxygenase-derived products such as prostaglandins and leukotrienes from arachidonic acid [17, 18]. Also, sulfasalazine may suppress B-cell function and inhibits cytokine release such as TNF-*α* IL-1, IL-2, IL-6, IL-12, and IgM and IgG production [17]. Current chemical drugs used for the treatment of UC are associated with many side effects. Recent complementary medicines, especially herbal remedies, are used increasingly by patients with inflammatory bowel conditions and have been reported to be effective [19–22]. *Quercus brantii* (QB) also known as Oak, from the Fagaceae family, is widely grown in Zagros mountainous region in the west and southwest of Iran [23, 24]. QB extract is rich in polyphenols, including tannin, gallic, and ellagic acid, which have antioxidant and anti-inflammatory properties [25]. Also, a significant amount of K, Fe, and Zn in fruit and leaves of QB was discovered [26]. Based on the results of the previous studies, the hydroalcoholic extract of QB have anti-inflammatory, antinociceptive, and homeostatic stabilizing effects. Another study showed that the gall of *Quercus brantii* Lindl. can exert antioxidative and anti-inflammatory effects on the biochemical and pathological parameters of colitis [27]. Furthermore, QB ethanolic and methanolic extracts have been shown to have antimicrobial and antioxidant properties [28–30]. Evaluation of *Quercus* in HT-29 cells showed a decrease in the levels of the inflammatory markers COX-2 and IL-8 by modulating the expression of NF-*κ*B [31]. The other *in vitro* studies confirmed the reduction of inflammatory cytokines and inhibition of lipopolysaccharide-induced production of TNF-*α* [32].

As far as our literature survey revealed, no studies have been reported so far on the potential application of QB hydroalcoholic extract on UC. Considering the multiple beneficial effects of QB in the literature, this study was aimed to evaluate the effects of QB extract on UC *in vitro* and *in vivo.* The preventive potential and antioxidant capacity of the QB was also evaluated. Moreover, to confirm the potency of treatments, histopathology and macroscopic were assessed.

## 2. Methods

### 2.1. Chemicals and Reagents

2,4,6-trinitrobenzene sulfonic acid (TNBS), carboxymethyl cellulose (CMC), sulfasalazine, and ethanol were purchased from Merck chemical company (Germany). Shiraz University of Medical Sciences (SUMS) supplied the laboratory facilities, including enzyme-linked immunosorbent assay (ELISA) kits for detecting TNF-*α*, IL-6, and NO. All other chemicals reached analytical grade.

### 2.2. Ethical Considerations

This experimental study was designed in Shiraz University of Medical Sciences to assess the effects of QB extract in Wistar rats with TNBS-induced ulcerative colitis. All animal experiments were conducted according to the guidelines approved by the SUMS Ethics Committee (No. 91-01-36–4560) and the Animal Research Reporting in Vivo Experiments guidelines (ARRIVE). Plant collection, storage, and hydroalcoholic extract preparation were following international legislation and the guidelines of the Pharmacognosy Department of Shiraz University of Medical Sciences, Shiraz, Iran. At the end of the experiment, the colon tissues were harvested and deepen in buffered formalin, stored for stereological evaluations. Rats were euthanized with a rapid and humane method using a 30% volume displacement rate of CO_2_ increases above 70% in the induction chamber.

### 2.3. Study Design and Selection of Animals

Seventy male Wistar rats, weighing 200 ± 20 g, were purchased from the Laboratory Animal Center of SUMS. The animals were kept in pathogen-free conditions at a constant temperature of 23 ± 2°C with a relative humidity of 55 ± 5%, on a 12 hrs light/dark cycle, and with a balanced diet and free access to water. The rats were fed adaptively in new surroundings for several days before the beginning of the experiment [33].

### 2.4. Plant Material and the Preparation of the Extract

QB fruits were purchased from available markets in Fars province, Iran (Herbarium No. 4179), and authenticated by a taxonomist of the Pharmacy School, Shiraz University of Medical Sciences (SUMS). To prepare the extract, the dried fruit barks of the plant were separated and powdered by an electric blender. 100 g of the bark was extracted by percolation with 70% ethanol at room temperature. The process was continued three times, and then, it was filtered and evaporated under reduced pressure to obtain a dry residue [34].

### 2.5. Gel Preparation

The gel-forming agent was prepared based on the previously reported procedure [35]. Briefly, 5% glycerol was added to 2% sodium carboxymethyl cellulose (NaCMC) and continuously stirred with a mixer. Next, *QB* extract was dissolved in deionized water and the mixtures were gradually added to the glycerol–NaCMC. Finally, the prepared gel was homogenized for 30 minutes and the prepared gel was collected in an aluminum tube in the refrigerator [36].

### 2.6. Induction of Colitis

Experimental colitis was induced in rats according to a method suggested in a published article [37]. Shortly, 24 hrs fasted rats were lightly anesthetized with ether; then, a 2-diameter polypropylene catheter was carefully inserted 8 cm through the rectum into the colon. A solution of 2,4,6-trinitrobenzenesulfonic acid (TNBS, 150 mg/kg) was dissolved in 50% (V/V) ethanol to break the intestinal barrier and was slowly injected into the colon while the control group received only 50% ethanol. Animals were hanged in the air vertically for 40 seconds and then returned to their cages [38].

### 2.7. Experimental Animals and Procedures

Animals were divided into seven equal groups, each of them having ten rats. The groups were classified as follows:

Group 1: Sham control group received normal saline orally
Group 2: Negative control received vehicles (TNBS)
Group 3: They received 200 mg/kg of the QB extract orally
Group 4: They received 400 mg/kg of the QB extract orally
Group 5: They received 200 mg/kg of the gel QB extract rectally
Group 6: They received 400 mg/kg of the gel QB extract rectally
Group 7: They received 500 mg/kg of sulfasalazine orally

All the treatments were administered 12 h after TNBS instillation and continued daily for six consecutive days. The sham control group received normal saline in an equal volume. All treatment regimens were given once a day for six days. During the therapy, body weight, stool appearance, visible stool, and occult bleeding were tested daily [39]. On the last day of the experiment (7th day), blood samples were collected to measure the levels of different inflammatory cytokines. At the end of the experiments, the colon was removed from rats, a 10 cm distal segment of the colon was dissected and flushed with cold saline. The dissections were weighed and cut open to be used for further macroscopic and histological investigation.

### 2.8. Assessment of Inflammatory Mediators

Measurements of inflammatory mediators, including TNF-*α* (Diaclone, France, Batch No: 3100–41), IL-6 (ZellBio GmbH, Ulm, Germany, Batch No: ZB-OER11017815), and NO (ZellBio GmbH, Ulm, Germany, Batch No: ZB-A81925) were performed. Blood samples were collected and centrifuged at 4000 RPM for 20 min to obtain the serum for the assessment of cytokines. The assays were carried out according to the manufacturer's instructions with some modifications. The details of the method were reported in our previous study [21].

### 2.9. Assessment of Colonic Myeloperoxidase (MPO)

MPO (KiaZist, Bahar, Hamadan, Iran, Batch No: 0076) activity in the soft tissue of the colon was measured as was described by Krawisz et al. [40]. The samples were weighed, shredded, and homogenized in 10 ml of cold 50 mmole potassium phosphate buffers, pH 6.0, which contained 0.5% hexadecyl trimethyl ammonium bromide (HETAB), and 10 mm ethylenediaminetetraacetic acid (EDTA). Then, the homogenates were sonicated and centrifuged at 2000 RPM for 20 min so that the final supernatant liquid could be used for MPO assay by ELISA kits. The results were expressed as units/mg colonic tissue.

### 2.10. Assessment of Malondialdehyde (MDA) as Lipid Peroxidation

As described by Ohkawa et al., the MDA (ZellBio GmbH, Ulm, Germany, Batch No: A317725) level (nmol/g wet tissue) is a factor that indicates lipid peroxidation. The increased level of MDA, caused by inflammation, reflects a high thiobarbituric acid (TBA) value [41]. The compound of TBA reaction with lipid peroxidation can be detected by spectrophotometrically (TBARS). Thus, the increased level of TBARS can be considered as an established indirect measure of the oxidation of polyunsaturated free fatty acids, as the principal end product of tissue inflammation and cell lysis.

### 2.11. Macroscopic Scoring

To assess macroscopic damage, the removed colons were scored using a 4-point numerical scale ranging from 0 to 4, where 0 indicates no macroscopic changes, 1 indicates only mucosal erythema, 2 indicates mild mucosal edema, slight bleeding, or small erosions, 3 indicates moderate edema, slight bleeding ulcers, or erosions, and 4 indicates severe ulceration, edema, and tissue necrosis [42]. As in previous similar studies, the ratio of wet tissue weight to the length of the colon was calculated to assess the intensity of edema [41, 43].

### 2.12. Histopathological Study

A blinded expert pathologist of the SUMS evaluated and categorized the tissues based on a 0–4 scale of inflammation, in which inflammatory response was graded (0) when there was no inflammatory cell in lamina propria, (1) when there were only scanty cells in lamina propria, (2) when there were countable inflammatory cells in lamina propria, (3) when there were both marked leukocyte infiltration and increased vascularity and edema, and (4) when there were signs of ulceration, sloughing, and/or transmural infiltration [44].

### 2.13. Statistical Analysis

Statistical analysis was performed with SPSS statistical software (Version 19.0, Chicago, IL, USA). The results are expressed as mean ± standard error of the mean (SEM). Kolmogorov-Smirnov test was used for the assessment of normality. Analysis of variance (ANOVA), followed by an appropriate post hoc test (Bonferroni's test), and the Kruskal-Wallis test were used for data analysis. *P* values less than 0.05 were considered statistically significant.

## 3. Results

### 3.1. Plant Extract

The percentage yield of crude ethanolic extract of QB was 9.8 g (9.8% yield, w/w concerning the dry plant material). Doses equivalent to 200 and 400 mg of the dried extract per kg body weight were calculated and then suspended in 0.5% CMC solution (as a vehicle) for the experiment.

### 3.2. The Effects of QB Extract on Body Weight, and Gross Appearance of TNBS-Induced Colitis

**Figure 1:**
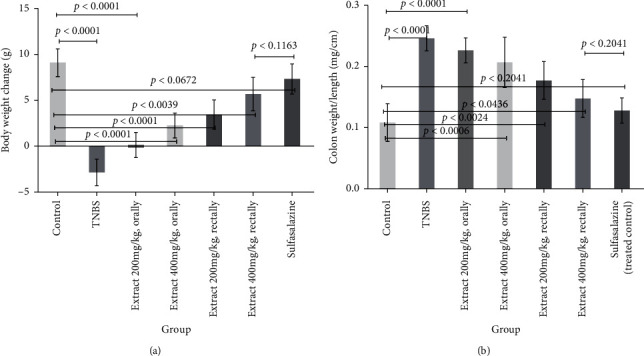
Effects of QB extract administration or sulfasalazine on body weight changes (a) and colon weight/length ratio in rats of TNBS experimental colitis (b). The *p* < 0.05 indicates a significant difference.

As compared to the sham group, after 24 hours, the TNBS-induced rats with acute colitis had hypomotility, gross bleeding, purulent stool, or diarrhea and their body weight decreased significantly. On the second day, sulfasalazine and different doses of QB extracts were administered orally or rectally for six consecutive days. All treated rats, except the rats treated for TNBS-induced colitis, showed improvement in the symptoms mentioned above. The changes which occurred in the bodyweight on the seventh day are reported in Figure 1. No significant difference was observed on body weight between the 400 mg/kg extract-treated rats and the sulfasalazine-treated group (*P* < 0.1163).

The bodyweight in all treated rats recovered significantly compared with that in the sham group, especially the groups which received 500 mg/kg sulfasalazine and 400 mg/kg of QB extracts. Notably, no significant difference was observed between the 400 mg/kg treated rats and the sulfasalazine-treated group (*P* < 0.2041).

### 3.3. The Effects of QB on Macroscopic and Histological Changes in the Colon

**Figure 2:**
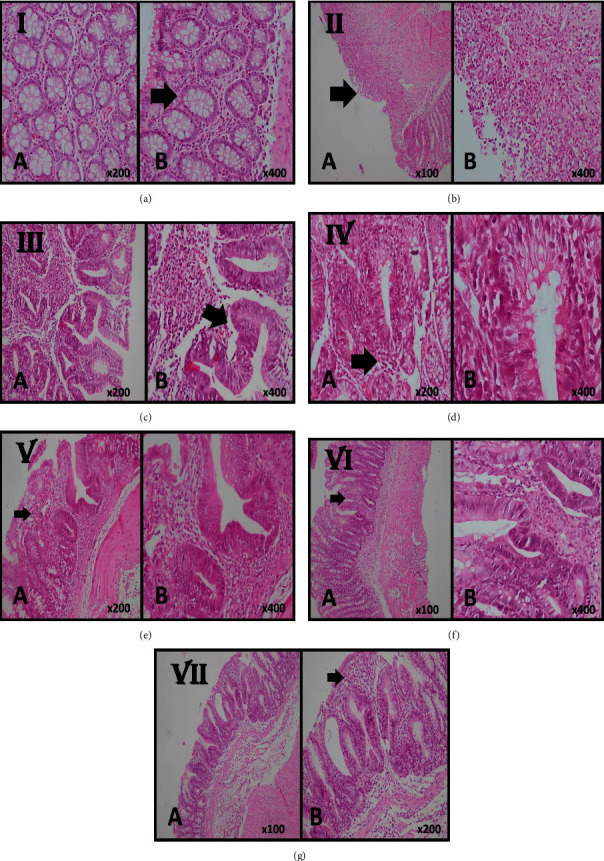
Histological sections of colonic mucosa in case and control groups. (a) Normal mucosa with the intact epithelial surface as well as normal colonic crypts (arrow) in the sham control group (A, B H & E stain, ×200, ×400). (b) In the negative control group, the slide shows TNBS-induced colitis with severe destruction with surface ulceration (arrow) and severe inflammatory cell infiltration representative of chronic colitis with severe activity, (A, B H & E stain, ×100, ×400). (c) Colonic mucosa of the cases who received 200 mg/kg QB extract orally shows moderate infiltration of inflammatory cells in lamina propria and mild crypt destruction and loss of goblet cells and cryptitis (arrow) representative of chronic colitis with mild activity (A, B H & E stain, ×200, ×400). (d) Colonic mucosa of the cases who received 400 mg/kg QB extract orally shows mild infiltration of inflammatory cells in lamina propria (arrow) and no crypt destruction and with few crypts with loss of goblet cells (A, B H & E stain, ×200, ×400). (e) Colonic mucosa of the cases who received 200 mg/kg QB extract rectally shows mild infiltration of inflammatory cells in lamina propria (arrow) and no crypt destruction and with few crypts with loss of goblet cells (A, B H & E stain, ×200, ×400). (f) Colonic mucosa of the cases who received 400 mg/kg QB extract rectally shows near-normal colonic mucosa with few inflammatory cell infiltrates in lamina propria (arrow) and focal crypts with loss of goblet cells (A, B H & E stain, ×100, ×400). (g) Colonic mucosa of the treated rats with 500 mg/kg of sulfasalazine shows near-normal colonic mucosa with focal crypts with loss of goblet cells with few inflammatory cell infiltrates in lamina propria (arrow) (A, B H & E stain, ×100, ×200).

In the next step, the gross appearance was evaluated in the laboratory. TNBS-induced models revealed severe edema, inflammation, and hyperemia of the colon in comparison with the sham control group, which had no or small inflammation. As shown in Table 1, the six-day administration of QB extracts and sulfasalazine significantly improved the colon macroscopic scores and decreased hyperemia and inflammation of the colon. More pronounced improvement was observed in the group which received 400 mg/kg of the extract rectally; this improvement was comparable with what was observed in the group which received sulfasalazine. It is worth noticing that no cytotoxicity was observed in the treated groups. The histological features of TNBS-induced models included edema, loss of goblet cells, loss of mucosal architecture and epithelium, ulceration of mucous, acute inflammatory cell infiltration, and thickening of colon lamina propria (Table 1). Treating rats with sulfasalazine (500 mg/kg) and different doses of QB extracts (200 and 400 mg/kg) could meaningfully limit the extent and attenuate the severity of the histological effects of TNBS-induced cellular damage. These treatments, i.e., sulfasalazine (500 mg/kg) and different doses of QB extracts (200 and 400 mg/kg), also improved the crypt architecture and edema of the colon. These changes were more prominent in rats that received sulfasalazine and 400 mg/kg of the QB extract rectally than in other rats. The histological changes which occurred after the induction of TNBS colitis and the effects of the treatments are shown in Figure 2.

### 3.4. The Effects of TNBS Induction and Treatments (QB Extracts and Sulfasalazine) on Cytokines Secretion

**Figure 3:**
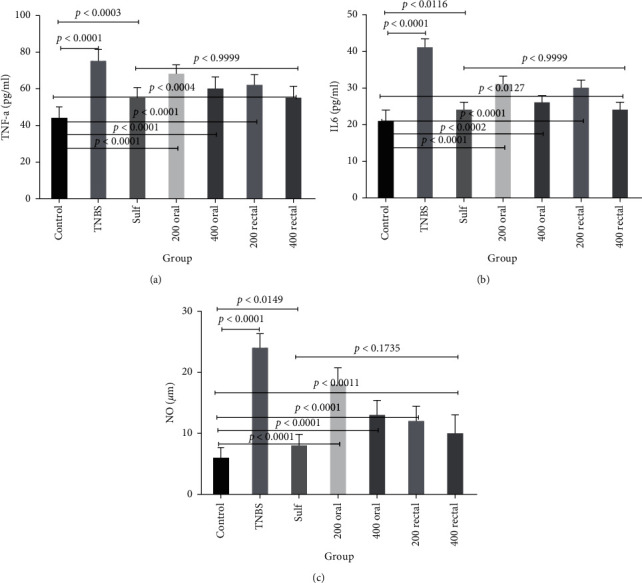
The effects of oral and rectal administration of different doses of QB extracts and sulfasalazine on TNF-*α* (pg/ml serum) (a), IL-6 (pg/ml serum) (b), and NO (*μ*m) (c) in rats with TNBS-induced colitis. Data are expressed as means ± SEM. The *P* < 0.05 indicates a significant difference.

Untreated TNBS-induced control rats had higher serum levels of TNF-*α* in comparison with the rats in the sham control group (75 ± 6.03 vs. 44 ± 5.66 pg/ml, respectively, *P* < 0.0001). After six days of treatment, the level of TNF-*α* improved in the sulfasalazine (*P* < 0.0003) and QB-extract-treated groups (Figure 3(a)). As displayed in Figure 3(b), the same patterns of increase and decrease were observed for serum IL-6 levels (an increase of 41 ± 2.21 vs. a decrement of 21 ± 2.75 pg/ml, *P* < 0.0001). Serum NO concentration was notably higher in the TNBS-induced control group compared with that of the sham (24 ± 2.17 vs. 6 ± 1.59 *μ*m, respectively, *P* < 0.0001) while groups treated with QB extracts and sulfasalazine had lower serum NO concentration compared to the nontreated TNBS-induced group (Figure 3(c)), suggesting that QB extract could inhibit the release of this inflammatory cytokine. Interestingly, no significant difference was observed between the 400 mg/kg QB-treated rats and the sulfasalazine-treated group (*P* < 0.173). The anti-inflammatory effects of the QB extracts (200 or 400 mg/kg, orally, or rectally) were confirmed in this study. The decreases observed in the levels of inflammatory cytokines were all statistically significant in the treated group compared to those in the nontreated TNBS-induced group. The TNF-*α* IL-6, and NO levels improved more significantly in the rats treated with QB extracts at a dose of 400 mg/kg via the rectal route compared to those in other rats; however, there were no statistically significant differences between the rats treated with 400 mg/kg of QB extracts via the rectal route and those treated with sulfasalazine in terms of the TNF-*α*, IL-6, and NO levels.

### 3.5. The Effects of QB Extract on Colonic MPO

**Figure 4:**
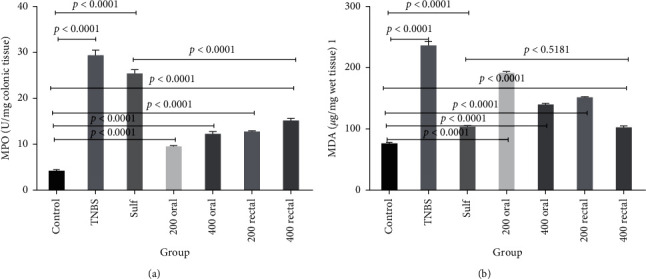
The effects of different doses of QB extracts and sulfasalazine (500 mg/kg) on the colonic MPO activity and MDA level in rats with TNBS-induced colitis. Data are expressed as means ± SEM. The *P* < 0.05 indicates a significant difference.

As an acute inflammatory biomarker, the MPO enzyme is mostly found in neutrophils, and its activity indicates the degree of neutrophil infiltration [45]. The MPO activities in the homogenates colon of all groups were measured and the results can be seen in Figure 4(a). The MPO activity in the TNBS-induced control group was significantly higher than that in the sham control group (29.38 ± 1.02 vs. 4.24 ± 0.25 U mg^−1^ colonic tissue, respectively, *P* < 0.0001). Although the MPO activity decreased in the groups treated with sulfasalazine and QB extracts (200 and 400 mg/kg, orally and rectally), a normal value, equal to that in the sham control, was not achieved in any of these treated groups. In this investigation, the rectal administration of QB extract was observed to have a better inhibitory effect on MPO compared to sulfasalazine as the positive control (*P* < 0.0001).

### 3.6. The Effects of QB Extract on Cell Lipid Peroxidation

The extent changes of lipid peroxidation in the homogenates bowel of all groups were measured, and the results are presented in Figure 4(b). As compared with that of the sham control group, the MDA level significantly increased in the TNBS-induced control group (236.35 ± 5.78 vs. 76.41 ± 1.64 *μ*g mg^−1^wet tissue, respectively, *P* < 0.0001) whereas it significantly decreased in the treated groups (QB extracts (200 and 400 mg/kg, orally, and rectally) and sulfasalazine). The decrease observed in the level of MDA was comparable between the sulfasalazine and QB-extract-treated groups, and there is not significant dereference between sulfasalazine and 400 mg/kg QB extract rectally (*P* < 0.5181).

## 4. Discussion

The major goal of this study was to assess the effects of QB extract in an animal model of TNBS-induced UC. The findings of this study revealed that the QB hydroalcoholic extract was beneficial in treating TNBS-induced colitis by reducing oxidative states, inflammations, and hemostatic instability.

UC, as a significant type of IBD, is a common illness with various degrees of inflammation, colorectal involvement, and intensity of occurrence. Colon cancer is more prevalent in patients with UC than in a healthy population [46, 47]. Furthermore, the social and economic impacts of this disease could affect UC patients' quality of life [48]. The treatment of IBD is extremely difficult due to the heterogeneity in this disease etiology, pathology, and clinical presentations. The available treatments have been reported to have different efficacy and side effects; some of these side effects are serious, especially during or after lengthy consumption [49, 50].

Sulfasalazine is a common drug used in the treatment of IBD. However, sulfasalazine therapy can cause adverse reactions, including idiosyncratic, skin reactions, hepatitis, pneumonitis, or hematologic side effects and reduced male fertility. Also, the sulfapyridine moiety of sulfasalazine results in toxic effects including headache, nausea, anorexia, and dyspepsia which limit the usage of sulfasalazine. Due to side effects, limited dosing (no greater than 3 g/day) was allowed to administer. On the other hand, sulfasalazine has a minimal immunosuppressive effect due to only 4-5 h a half-life. Therefore, UC treatment is highly necessary especially with those agents which are not producing side effects such as medicinal plants [51]. Herbal medicine is believed to be relatively safe, efficient, available, and less expensive than conventional medicine. Hence, they are more popular among patients [52]. Based on the literature review, some natural products were introduced with anti-UC effects. El-Abhar et al. [53] evaluated the modulating effects of ginger extract on the extent and severity of UC in rats; they found out that ginger extract (GE) had valuable and beneficial effects, comparable with those of sulfasalazine [53]. Han et al. suggested that *Cordyceps militaris* extract had a downregulatory effect on the production and expression of inflammatory mediators from macrophages, which was found to suppress acute-induced colitis in mice. Accordingly, *Cordyceps militaris* extract can be applied as an agent to prevent or treat IBDs [54]. After that, He et al. showed the anti-inflammatory effects of Pulvis Fellis Suis extract in mice with UC. They hypothesized that the Fellis Suis extract could have anti-inflammatory effects by possibly reducing the production of upregulated TNF-*α* and IL-6 [55].

Moreover, Han et al. conducted two experimental models of induced colitis, i.e., dextran sulfate sodium (DSS) in mice and 2, 4-dinitrochlorobenzene (DNCB) in the rat. The results showed that treatment with Suqingwan watered pills could reduce colon injury [56]. However, the assessment of inflammatory cytokines was not included. Takhshid et al. evaluated the potential healing effects of licorice extract on acetic acid-induced UC in rats in a controlled study. [57].

There are limited reports about the effect of QB and its derivatives in humans and animals and there are no reports about its effects in UC.

Acorn, the fruit of Oak (Quercus tree) has been reported to contain vitamins, nutrients, carbohydrates, and minerals. It also contains considerable amounts of phenolic, tannin, catechin, epicatechin, and gallocatechin components [58, 59]. Regarding the role of microbes and the oxidative inflammatory process in inducing colitis, the initial inferences could be drawn based on the beneficial effects of the antibacterial, antioxidant, and anti-inflammatory properties of QB [25, 27, 60].

In the study on the dietary Persian oak (*Quercus brantii* var. *persica*), Bohlouli et al. [61] reported that the oak fruit extract induced a significant increase in the immunological and hematological parameters but had no effects on survival and growth [61]. Azizi et al. showed the dose-dependent accelerated healing effects of QB active components (tannin = 8.2%) on gastric ulcers, a finding which supports the traditional use of QB [34]. Another strength of this study is the use of hydrogels from CMC. CMC is commercially available water-soluble cellulose with biocompatibility and nontoxicity, which are compulsory conditions for polymers used in biomedical applications. [62]. CMC can absorb and retain a huge proportion of water in the interstitial sites due to their structures. It also keeps and increases the homogeneous and stable mixture of QB extract for several days and improves the interaction between extract and the target tissue [36].

It has been reported that proinflammatory cytokines such as IL6 and TNF-*α* and inflammatory mediators such as NO rise in primary stages of TNBS-induced colitis in rats. The current study demonstrated the significant dose-dependent reduction of these cytokines in rats treated with QB extract which is close to the positive control and sham groups. The fact that the exact mechanisms of *QB* improve biochemical and stereological parameters of UC is not clear. The high potency of QB may be due to high phenolic acids (gallic acid, ellagic acid) and flavonoids (quercetin, catechin, naringin) compounds [63]. In detail, gallic, ellagic acid, and quercetin as the most common antioxidant compounds found in QB decreasing damage to proteins, lipids, and macromolecules and moderately increase the total antioxidant capacity of plasma on human subjects. This also supports the anti-inflammatory effects of QB extracts [63]. Studies have suggested that gallic acid and phenolic compounds exert potentially clinically useful anti-inflammatory effects mediated through the suppression of p65-NF-*κ*B and IL-6/p-STAT3 (Y705) activation [64]. The antibacterial and antifungal activity of QB was also well documented considering the fact that abnormal bacteria can cause inflammation through the impairment of epithelial cell metabolism in UC. It is also suggested that the second mechanism of QB can be related to the antimicrobial properties of the mentioned extract [65]. The other study suggested that gallic acid treatment could modulate the microbiota composition towards a similar proportion to the control group [66].

The other compound in QB was quercetin. Studies on phytoestrogens about bone health in postmenopausal women and prostate health in men have shown promising results. Quercetin significantly inhibited TNF-*α* production and gene expression in a dose-dependent manner [67]. Also, it significantly attenuates LPS-induced production of TNF-*α* and IL-1*β* in macrophages. It also causes a significant reduction in the phosphorylation and degradation of inhibitor of *κBα* and decreasing the nuclear level of nuclear factor-*κ*B (NF-*κ*B) and NF-*κ*B binding activity [68].

Khavandi et al. investigated the effect of *QB* in a murine model of colitis induced in rats. They concluded that *Quercus brantii* induced antioxidants especially via inhibition of inflammatory cytokines such as TNF-*α*, IL-1*β*, and MPO. This might relate to a high amount of total phenols (88.43 ± 7.23/100 g of sample) and free gallic acid (3.74% of dry weight) [27]. Recently, Castejón et al. showed that *Quercus ilex* improves TNBS-induced colitis via MAPKs/NF-*κ*B inhibition and Nrf2/HO-1 activation signaling pathways. This in return results in reducing the production of Th1 proinflammatory cytokines and COX-2 and iNOS overexpression [69].

Considering that the increase in two oxidative enzymes, namely, MAO and MBA, correlates with high inflammation and their reduction with improved symptoms and inflammation, it can be stated that QB extract had antioxidant effects in this study. Histological pieces of evidence indicated a significant improvement in the microscopic features, crypt architecture, and colon edema in rats treated with the mentioned extract which highlights the healing effect of QB in UC. QB extract consumption also resulted in minor bodyweight loss in the treated group compared with the control groups. Also, acute inflammatory cell infiltration and the thickening of lamina propria were diminished. Regarding the biological evaluations of QB extract as a natural product on UC and various diverse effects of sulphasalazine discussed above, it can be seen that 400 mg QC rectally has even good potency compared to that of sulphasalazine. However, merit studies are required to clarify the pharmacological properties and mechanisms of QB and phenolic compounds in QB extract.

## 5. Conclusion

*Quercus brantii* medicinal plant extract showed high TNF-a, IL-6, and NO inhibitory activity, indicating that the bioactive compounds present in the extract have the potential to reduce inflammation. It has shown significant lowering activity of MAO and MBA. This result suggested the promising healing stimulatory and anti-inflammatory properties as well as antioxidant effects of QB which make it an appropriate agent for the treatment of UC. Further studies are required to evaluate its other beneficial properties and confirm its clinical effectiveness in humans.

## Figures and Tables

**Table 1:** Effects of oral and rectal administration of different doses of QB extract (200, 400 mg/kg) and sulfasalazine (500 mg/kg) on colonic macroscopic damage (A) and histological score (B) rats' TNBS-induced colitis.

Group	Macroscopic score	Histological score
Sham control	0.55 ± 0.11	0.78 ± 0.09
Negative control	4.97 ± 0.26^##^	13.1 ± 0.48^##^
QB extract 200 mg/kg orally	3.04 ± 0.21^##∗∗^	9.1 ± 0.37^##∗∗^
QB extract 400 mg/kg orally	2.17 ± 0.18^##∗∗^	7.95 ± 0.26^##∗∗^
QB extract 200 mg/kg rectally	1.88 ± 0.33^##∗∗^	7.23 ± 0.15^##∗∗^
QB extract 400 mg/kg rectally	1.43 ± 0.14^##∗∗^	6.51 ± 0.22^##∗∗^
Sulfasalazine (treated control)	1.24 ± 0.16^∗∗^	6.12 ± 0.41^##∗∗^

Data are expressed as means ± SEM. ^##^*P* < 0.01 vs. sham control; ^∗^*P* < 0.05, ^∗∗^*P* < 0.01 vs. TNBS control.

## Data Availability

The datasets used and analyzed during the current study are available from the corresponding author on reasonable request. We have presented all data in the form of tables and figures.
